# Supplementary material for: Craft: a machine learning approach to dengue subtyping
Source: Bioinform Adv. 2025 Oct 6;5(1):vbaf224. doi: 10.1093/bioadv/vbaf224 (PMC12527244; doi:10.1093/bioadv/vbaf224)
Supplement: vbaf224_Supplementary_Data [file vbaf224_supplementary_data.zip › Revised Supplementary Material.pdf]

Craft: A Machine Learning Approach to Dengue Subtyping

Supplementary Material

Table 1: Classification accuracy of all models on the different label sets (indicated by the four right-most column headings) based on the samples in the hold-out test set. The training set used for each Craft model is indicated in the "Training Labels" column.

**Model**	**Training Labels**	**Genome Detective**	**GLUE**	**NextClade**	**Consensus**
Genome Detective	-	1.000	0.734	0.976	0.991
GLUE	-	0.734	1.000	0.730	0.744
NextClade	-	0.976	0.730	1.000	0.986
Craft	Genome Detective	0.988±0.001	0.734±0.001	0.976±0.001	0.986±0.002
Craft	GLUE	0.748±0.001	0.958±0.001	0.745±0.001	0.757±0.001
Craft	NextClade	0.973±0.001	0.729±0.001	0.996±0.001	0.983±0.001
Craft	Consensus	0.985±0.001	0.742±0.001	0.985±0.001	0.995±0.001

Table 2: Adjusted Rand Index (ARI) scores comparing lineage assignments between all methods. Higher scores indicate greater similarity in the clustering structure of lineage assignments.

**Method**	**Craft**	**GLUE**	**NextClade**	**Genome Detective**
Craft	1.000	0.673	0.982	0.977
GLUE	0.673	1.000	0.668	0.665
NextClade	0.982	0.668	1.000	0.967
Genome Detective	0.977	0.665	0.967	1.000

Table 3: Normalized Mutual Information (NMI) scores comparing lineage assignments between all methods. Higher scores indicate greater mutual dependence between assignment structures.

**Method**	**Craft**	**GLUE**	**NextClade**	**Genome Detective**
Craft	1.000	0.932	0.994	0.992
GLUE	0.932	1.000	0.930	0.928
NextClade	0.994	0.930	1.000	0.990
Genome Detective	0.992	0.928	0.990	1.000

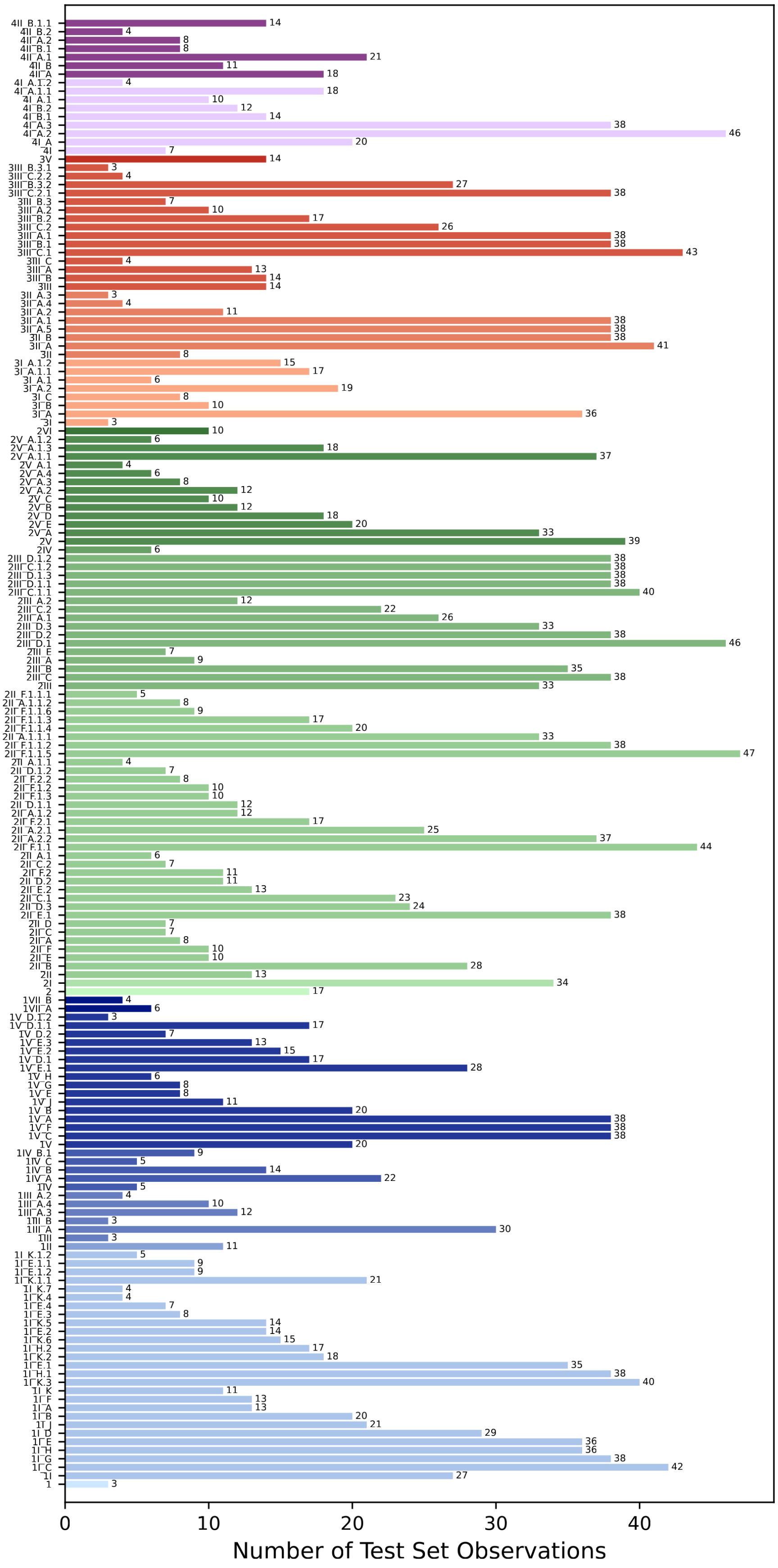

Figure 1: Distribution of test set class size.
